# Complex Large-Deformation Multimodality Image Registration Network for Image-Guided Radiotherapy of Cervical Cancer

**DOI:** 10.3390/bioengineering11121304

**Published:** 2024-12-23

**Authors:** Ping Jiang, Sijia Wu, Wenjian Qin, Yaoqin Xie

**Affiliations:** 1 Shenzhen Institute of Advanced Technology, Chinese Academy of Sciences, Shenzhen 518055, China; p.jiang@siat.ac.cn (P.J.); wusijia2017@sina.com (S.W.); wj.qin@siat.ac.cn (W.Q.); 2 University of Chinese Academy of Sciences, Beijing 100049, China

**Keywords:** cervical cancer, multimodality registration, deformation field, multi-level, wavelet transformation

## Abstract

In recent years, image-guided brachytherapy for cervical cancer has become an important treatment method for patients with locally advanced cervical cancer, and multi-modality image registration technology is a key step in this system. However, due to the patient’s own movement and other factors, the deformation between the different modalities of images is discontinuous, which brings great difficulties to the registration of pelvic computed tomography (CT/) and magnetic resonance (MR) images. In this paper, we propose a multimodality image registration network based on multistage transformation enhancement features (MTEF) to maintain the continuity of the deformation field. The model uses wavelet transform to extract different components of the image and performs fusion and enhancement processing as the input to the model. The model performs multiple registrations from local to global regions. Then, we propose a novel shared pyramid registration network that can accurately extract features from different modalities, optimizing the predicted deformation field through progressive refinement. In order to improve the registration performance, we also propose a deep learning similarity measurement method combined with bistructural morphology. On the basis of deep learning, bistructural morphology is added to the model to train the pelvic area registration evaluator, and the model can obtain parameters covering large deformation for loss function. The model was verified by the actual clinical data of cervical cancer patients. After a large number of experiments, our proposed model achieved the highest dice similarity coefficient (DSC) metric compared with the state-of-the-art registration methods. The DSC index of the MTEF algorithm is 5.64% higher than that of the TransMorph algorithm. It will effectively integrate multi-modal image information, improve the accuracy of tumor localization, and benefit more cervical cancer patients.

## 1. Introduction

Cervical cancer is a common malignant tumor in the female reproductive system, with an increasing incidence among younger age groups [1,2]. For patients with locally advanced and inoperable cervical cancer, the current effective treatment option is image-guided radiation therapy (IGRT) [3]. This technique delivers a high dose of radiation to the tumor while surrounding normal organs receive only a low dose. This increases the chances of a tumor cure while reducing the potential reactions and damage to normal tissues.

In a clinical setting, accurately locating the tumor area is crucial. Therefore, one of the key challenges in image-guided cervical cancer treatment is the task of image registration [4]. Computed Tomography (CT) has important clinical value for the dose planning of the radiotherapy program in the IGRT system. However, CT images are sensitive to bone information but have low soft tissue contrast, which limits their effectiveness in application. In order to avoid the side effects of radiotherapy treatment and reduce the damage of healthy organs, MR images can better display cervical lesions and endangered organs [5,6]. MR images can well distinguish soft tissue organs. By registering the fusion of two different imaging modalities, more information can be provided to effectively increase the dose rate in the target area while minimizing radiation exposure to organs at risk outside the tumor.

**Figure 1:**
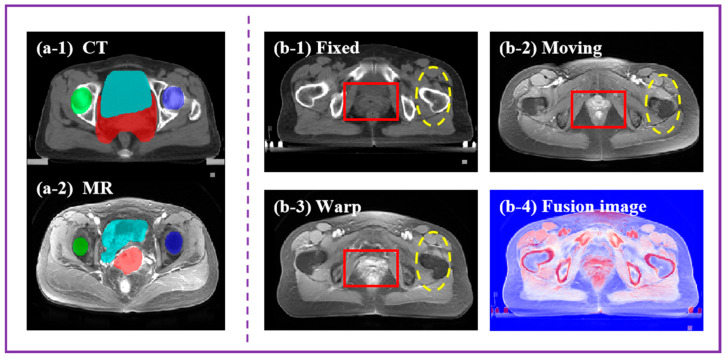
The CT and MR images of the same patient are shown in (**a**), and the large deformation can be found between the multimodality images, which also increases the difficulty of our algorithm design. (**a-1**) represents the patient’s CT image, and (**a-2**) represents the patient’s MR image. (**b-1**) shows the CT image and (**b-2**) shows the MR image. (**b-3**) shows the registration image, and (**b-4**) shows the superimposed image of the warp image and fixed image. The MTEF model can better learn the features of the fixed image, achieving high-precision multimodality image registration and fusion.

**Figure 2:**
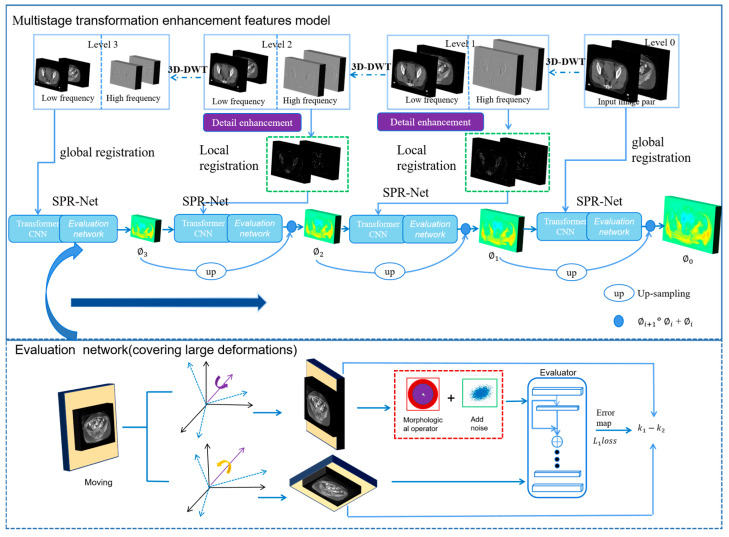
The proposed model architecture diagram.

Multi-modality image registration of the cervix has the problem of large-scale deformation (as shown in Figure 1(a-1,a-2)), even in the same patient, due to their own state and movement; the bladder and rectum have significant deformation, even discontinuous deformation. In recent years, with a large number of researchers joining the research of medical image registration, many image registration algorithms and related multimodality feature extraction algorithms [7,8] have been proposed. Examples such as elastic models [9], Demons [10], B-spline [11], SyN [12], etc. have achieved success in anatomy. These methods are all based on the optimizing energy function to complete the registration process, and these methods are computationally expensive and usually slow in practice [13].

Recently, image registration algorithms based on deep learning have become a research hotspot. Although deep learning will consume a lot of computing resources, it only takes a few seconds to register after training, and its performance is already better than that of traditional algorithms. At present, multi-modality image registration algorithms based on deep learning are mainly divided into supervised and unsupervised algorithms [14,15,16]. Among them, supervised deep learning registration algorithms need real ground-truth to guide model training; some ground-truth is synthesized, and others are generated by traditional registration methods. Yang et al. [17] proposed a supervised registration model that focuses on a large predictive deformation differential isomorphic metric mapping (LDDMM) model and promotes a block-by-block prediction strategy by predicting the momentum parameterization of LDDMM. Sokooti et al. [18] synthesized ground-truth by a deep learning method to estimate the displacement vector field directly from a pair of input images. However, the calculation cost of generating ground-truth is very high, and the registration effect of this kind of algorithm is seriously affected by the quality of ground-truth.

Therefore, the current research focus turns to unsupervised deep learning network algorithms that do not require ground-truth. Balakrishnan et al. [19], Wang et al. [20], and de Vos et al. [21] propose an end-to-end network that estimates deformable transformations by maximizing image similarity between image pairs without the need for ground truth values. The algorithm applies a similarity function (normalized cross-correlation (NCC) or cross-correlation (CC)) to train the model, the general representation of registration is learned from the training set, and a spatial transformation function is applied to warp the moving image. This algorithm has achieved good results in small deformation areas such as the head. However, the registration effect is not satisfactory in areas with more soft tissues and large deformation such as the pelvic area, and the training data in the pelvic area is relatively small, which is also a factor limiting the performance of the algorithm [22]. In recent years, transformer networks have become a popular focus in multimodal registration tasks, with models like the TransMatch [23] and TransMorph [24] models leading the field. These algorithms use transformers as encoders for feature extraction, which has proven effective for handling large deformation registration tasks. However, in complex regions where soft tissue organs intertwine, these transformer-based methods still need improvement when it comes to registering local organs accurately.

In order to solve the large deformation registration problem, Mok et al. [25] proposed a deep Laplacian pyramid image registration network (LapIRN), which addresses image registration optimization in a coarse-to-fine manner within the diffeomorphic mapping space. The model constructs a three-level image pyramid from the original image and uses a CNN to generate deformation fields from low to high resolutions at each level. Although LapIRN was designed for large deformation registration tasks, the model tends to get stuck in local minima, particularly in areas with a lot of soft tissue, such as the pelvis. Zhao et al. [26] recursively deform the moving images through stacked multi-level networks, ultimately aligning them to the fixed image. These methods bring a burden to the GPU, consuming significant computational resources, and the algorithm performance can be affected by accumulated errors during the process of model cascading. In addition, Cao et al. [27] proposed a region-adaptive deformation registration method for multi-modal pelvic image registration. In this method, the moving image and fixed image are generated by a multi-objective regression forest, the bone region is detected in the CT image and the soft tissue is detected in the MR region, and the whole registration process is driven by region detection. The number of key detection points gradually increases during the registration process. This method achieves good results in the case of small deformation, but in the case of large deformation, the accuracy decreases. Additionally, the algorithm complicates the entire registration process and heavily consumes computational resources.

In order to overcome the large deformation registration problem of multimodality images, we propose a multistage transformation enhancement features (MTEF) image registration network. This method utilizes wavelet transforms to extract different image components as inputs and proposes a shared pyramid registration network (SPR-Net) model to predict the registration deformation fields from coarse to fine. In addition, to improve registration accuracy, we trained the model with high-precision similarity measurements focused on the pelvic region (as shown in Figure 1(b1–b4), the MTEF algorithm can balance global and local feature registration).

## 2. Methods

Given a pair of input images, fixed image *I* and moving image *M*, the purpose of registration is to obtain a deformation field ϕ so that warped image M∘ϕ and image *I* are spatially and structurally aligned. In order to cope with the large deformation of the pelvic region registration work, we propose a multimodal image registration network based on the multistage transform enhancement feature (MTEF). Figure 2 shows the framework diagram of the MTEF algorithm, which mainly includes three parts: (1) image multi-resolution transform input; (2) shared pyramid registration network (SPR-Net); and (3) high-precision spatial evaluator network. We use wavelet transform to obtain different information of the image and further input the proposed SPR-Net registration network, which uses two independent Swin Transformers to extract different modal features. The deformation field is obtained by using the progressive prediction strategy at the decoder stage. At each stage of the decoder, the deformation field of the previous layer will be fused to gradually solve the problem of large deformation in complex areas. In addition, we use a deep learning network to obtain an accurate similarity measurement. The proposed model is registered four times in total, and the deformation fields of different stages are continuously integrated to further improve the local and global registration performance. This will be described in detail below:

### 2.1. Image Multi-Resolution Input

Wavelet transform can extract different components of images, and combination wavelet transform with deep learning can avoid the insufficient ability of the deep learning network to extract features in the case of small samples of data and improve registration accuracy. Wavelet theory has been applied in image synthesis and super-resolution reconstruction, but there is still limited research on its combination with deep learning in registration. The wavelet transform in the process of image decomposition will produce more components, leading to the question of which components to use and how to use them, which is also one of the focuses of this paper.

Wavelet transform extracts high-frequency component F(i) and low-frequency component X(i) from the original image Y(i), and the formula is as follows:

(1) Y(i)=X(i)+F(i)

**Figure 3:**
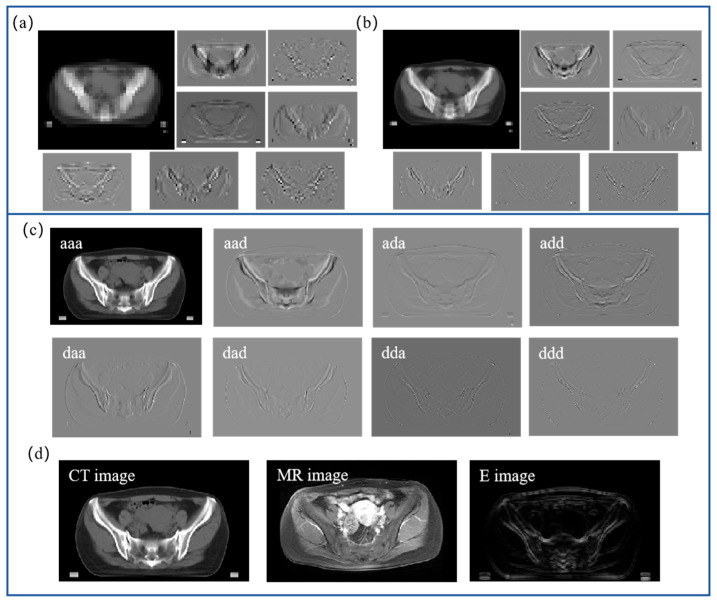
(**a**) In the third stage, eight sub-images are obtained after the decomposition of the CT image. (**b**) In the second stage, eight sub-images are obtained after the decomposition of the CT image. (**c**) In the first stage, eight sub-images are obtained after the decomposition of the CT image. (**d**) The patient’s raw CT and MR images and enhanced processing of high-frequency components.

The high-frequency component contains not only noise but also texture information. After wavelet decomposition, the high-frequency component F(i) can be decomposed into seven components, and the formula is as follows:

(2) $$F(i)=\left\{aad,ada,add,daa,dad,dda,ddd\right\}$$

where aad, ada, add, daa, dad, dda, and ddd represent different components in the high-frequency information. For a pair of a fixed image *I* and a moving image *M*, different components extracted by the wavelet transform algorithm are input into the MTEF algorithm separately, which makes algorithm use different scale and multi-level wavelet component information. Figure 3 shows the images at three stages of wavelet decomposition. Figure 3a,b are the decomposed sub-images of the first and second stages, Figure 3c is the first level of seven images, and Figure 3d is the enhanced processing image. We apply the three-dimensional discrete wavelet transform (3D-DWT) algorithm to decompose images *I* and *M* into three levels of wavelet decomposition. After each level of decomposition, we obtain eight sub-images (seven high-frequency components and one low-frequency component), including *aaa*, *aad*, *ada*, *add*, *daa*, *dad*, *dda*, and *ddd*. The aaa represents the low-frequency component and contains images of global information. The remaining seven components represent the high-frequency components, which represent the local information of the image. *a* and *d* represent low-frequency and high-frequency filters, respectively. We fuse and enhance seven high-frequency components according to the fusion rule shown in Equation (3) and use them as the input to the model, which allows the model to focus on local region registration. Conversely, using low-frequency components and the original image as the input enables the model to concentrate on global region registration. Effectively utilizing different components allows the network model to simultaneously consider both global and local region registration. This is crucial for achieving the high-precision registration of significant deformations in the pelvic region.

(3) $$E=\nabla_{aad}+\nabla_{ada}+\nabla_{add}+\nabla_{daa}+\nabla_{dad}+\nabla_{dda}+\nabla_{ddd}$$

where ∇aad, ∇ada, ∇add, ∇daa, ∇dad, ∇dda, and ∇ddd represent different components of gradient computation in the high-frequency information. We select the high frequency of the first stage for enhancement processing. As shown in Figure 3c, the moving image is formed by seven high-frequency components after the 3D-DWT process, representing the detail information of the image. The seven components are enhanced and combined to obtain *E*, as shown in Figure 3d. Inputting *E* into the network model will prompt the model to pay attention to the local region of the image. The model will perform two local region registrations and two global region registrations, so as to ensure that the deformation field obtained by the model can take into account both global and local characteristics and improve the accurate fusion of multimodality data of cervical cancer patients.

### 2.2. Shared Pyramid Registration Network

**Figure 4:**
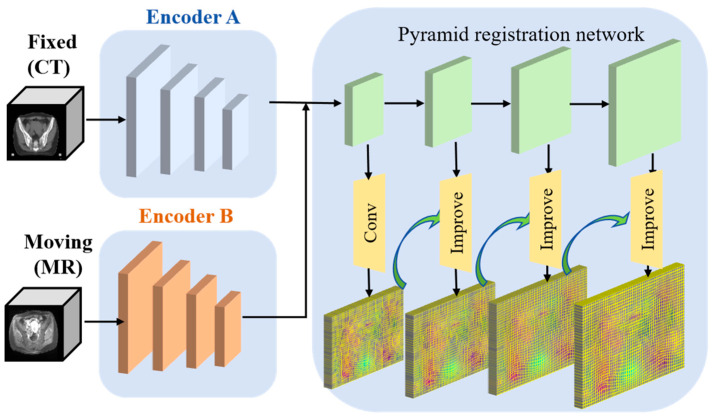
The architecture of this paper’s proposed shared pyramid registration network (SPR-Net). It includes two independent Swin Transformer encoders and a shared decoder. In the decoder part, we employ a pyramid registration strategy to predict the deformation field between images with large deformations, achieving a coarse-to-fine registration process.

After the 3D-DWT processing of fixed image *I* and moving image *M*, we can obtain the aaa component and enhanced component E of each image, and the MTEF algorithm will extract the global component twice and the local component twice. The pelvic region contains a large amount of soft tissue, making it difficult to distinguish between adjacent organs. Therefore, a model with strong feature extraction capabilities is required. However, most current multimodal registration algorithms use a single network for multimodal feature extraction, which is unable to effectively capture the different modal features. We propose a shared pyramid registration network (SPR-Net), which utilizes two independent Swin Transformer encoders to extract multimodal image features and a shared decoder to predict the deformation field (as shown in Figure 4). Swin Transformer [28] better understands the spatial correspondences in large deformations, allowing the model to focus on areas requiring deformation. Additionally, to address the challenge of registering large deformations, we employ a pyramid feature registration module in the decoder to predict the deformation field.

MTEF consists of four registration stages, the level3–level1 stages use a wavelet subband image as the input, and the level0 stage uses the original image as the input. For level3, the MTEF algorithm takes the low-frequency image aaa with the lowest resolution as the input (size 48×32×4) and extracts the coarse deformation field $\phi_{3}$ through the SPR-Net, which is also a global registration. The SPR-Net contains two independent Swin Transformer encoders to extract multimodal image features and a shared decoder to predict the deformation field. At level2 and level1, we use the high-frequency component enhanced image E after wavelet transformation as the input and use SPR-Net to obtain the deformation field $\phi_{2}$ (size 96×64×8); it is also a local characteristic deformation field. Upsample $\phi_{3}$ deformation field to the same size as $\phi_{2}$. The $\phi_{3}\circ \phi_{2}+\phi_{2}$ is taken as the final deformation field (size 384×256×32) of level2 [14,29]. Similarly, the same applies to the deformation fields for level1 and level0.

First, the model divides the fixed image and floating image into non-overlapping 3D blocks, the size of each block is K×K×K, and the total number of image blocks is $S=\frac{HWL}{K^{3}}$. Linear embedding maps them into one-dimensional vectors, and the planarization of each patch is denoted as token. The formula is as follows:

(4) $$I_{0}=[x_{p}^{1}F;x_{p}^{2}F;\cdots ;x_{p}^{N}F],F\in R^{p^{3}\times D}$$

The dimension of output $I_{0}$ is S×D. The D represents the embedding dimension of each image block. Since the registration process is a pixel-level task, different from the traditional Transformer network, it is not necessary to add position embedding. The network includes an encoder and decoder, and any spatial position difference between the output image and the fixed image will be reflected by the loss function and transmitted in reverse. Therefore, it is not necessary to add position embedding in the model; it also increases the complexity of the model. After linear projection, $I_{0}$ will undergo the patch merging of three stages and the Swin Transformer module of four stages. The patch merging module will splice features of each stage. Therefore, in the stage 2 stage, the feature output size becomes halved. That is, $\frac{H}{4}\times \frac{W}{4}\times \frac{L}{4}\times D\to \frac{H}{8}\times \frac{W}{8}\times \frac{L}{8}\times 2D$, and the output size of the last level is $\frac{H}{4}\times \frac{W}{4}\times \frac{L}{4}\times D\to \frac{H}{8}\times \frac{W}{8}\times \frac{L}{8}\times 2D\to \frac{H}{16}\times \frac{W}{16}\times \frac{L}{16}\times 4D\to \frac{H}{32}\times \frac{W}{32}\times \frac{L}{32}\times 8D$.

In addition to the patch merging layer, an important part of Swin Transformer is the alternating composition of 12 layers of multi-head self-attention (W-MSA) and multi-layer perceptron (MLP) blocks [28]. Unlike traditional Transformer networks, local self-attention computing is sufficient for an organ in the image and can save significant resources. Swin Transformer calculates self-attention within local windows (as shown in Figure S1a). When data enter the Swin Transformer block, multi-head self-attention within the window begins the calculation. To introduce connections between neighboring windows, Swin Transformer employs a shifted window design (as shown in Figure S1b). In order to cause the information interaction between the Windows, the offset window multiple self-attention (SW-MSA) is introduced. After the non-overlapping windows are divided in the *L* layer, the windows are re-divided in the *L* + 1 layer by offset half the window distance, so the information of some windows in different layers can be exchanged. Residual connections are used after each MSA and MLP to learn identity mappings and enhance the performance of the model. As shown in Figure S1b, after stage 1, the feature map is evenly divided into 2×2×2=8 windows, and then during stage 2, the number of windows becomes 3×3×3=27.

Pyramid Feature Registration Module: To address the challenge of multimodal large deformation registration, we employ a pyramid feature registration module in the decoder to predict the deformation field from coarse to fine. As shown in Figure 4, its structure is connected to the encoder via skip connections, with each layer containing an upsampling module to upsample the low-resolution deformation field, followed by a 3 × 3 × 3 convolution. In the first layer, deformation field $\phi_{m4}$ is obtained, which is upsampled using interpolation and applied to the high-resolution features of the moving image. Then, through a 3 × 3 × 3 convolution, the deformation field B is obtained in the second layer. This process is repeated iteratively until the final deformation field $\phi_{m1}$ is produced. The equation is expressed as:

(5) $$\phi_{m}=conv_{m}(f_{m}^{M}\circ up(\phi_{m-1}),f_{m}^{F})$$

where $conv_{m}$ represents the 3 × 3 × 3 convolution in the decoding layer, ∘ represents the function applied to the moving image, $f_{m}^{M}$ represents the moving image features at *m* layer, and $f_{m}^{F}$ represents the fixed image features at *m* layer. From the lowest resolution to the highest resolution, we obtain an increasing deformation field: $\phi_{m4}$, $\phi_{m3}$, $\phi_{m2}$, $\phi_{m1}$.

The design rationale is that while the deep features of different modal images are similar, their shallow features differ. Therefore, we utilize two independent Swin Transformer encoders and a shared CNN decoder. This design allows for progressively refining the estimated deformation field. For large deformation registration tasks, we adopt a Transformer structure with a larger receptive field and a pyramid strategy to gradually optimize the deformation field, aggregating high-level information and shallow details. This model can handle large deformations in multimodal images without distorting the local anatomical structures.

### 2.3. Similarity Measurement Evaluator

**Figure 5:**
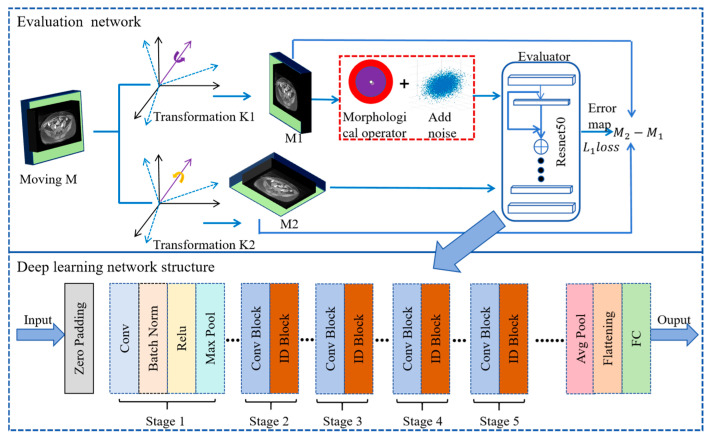
High-precision similarity measurement by deep learning combined with bistructural morphology.

The multi-modality image registration requires precise similarity measurement; the similarity operators based on traditional methods cannot meet the requirements of complex and large deformation pelvic region registration. In order to improve the registration accuracy, we created an accurate multimodality spatial evaluator to measure the spatial difference between two images; the model added bistructural morphology to the deep learning network to improve the algorithm’s evaluation of the difference between large deformed organs. The bistructural morphology uses two structural elements (such as different sizes), which can simultaneously capture multi-scale or heterogeneous characteristics of the image and improve the description ability of complex structures. The bistructural structure morphology and add noise processing were used to input a deep learning network to simulate the large deformation of the organ so as to further obtain comprehensive and accurate similarity parameters as the loss function. Figure 5 shows the algorithm structure diagram.

The specific scheme is to first perform two random space transformations, K1 and K2, on the original image M to obtain the transformed images M1 and M2, respectively. Spatial transformation operations include rotation, displacement, rescaling, and random pixel-by-pixel deformation. M1 and M2, from the same image M, differ only in spatial location. After M1 and M2 are generated, the spatial position error image is obtained by subtraction, which is used as the label for the training of the evaluator. Next, we apply bistructural morphology and add noise to M1 to create distribution differences between M1 and M2. Images with different spatial locations and distribution differences are fed into Resnet50 network models [30] to obtain similar measures covering large deformations.

**Figure 6:**
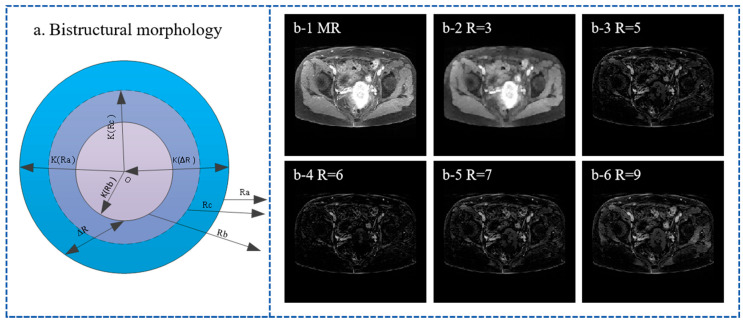
Bistructural morphology algorithm principle and image effect under different structure sizes. (**a**) represents the schematic diagram of the principle of bistructural morphology. (**b-1**) represents the MR image of the patient. (**b-2**) represents the processing effect with a radius of 3. (**b-3**) represents the processing effect with a radius of 5. (**b-4**) represents the processing effect with a radius of 6. (**b-5**) represents the processing effect with a radius of 7. (**b-6**) represents the processing effect with a radius of 9.

The dual-structure morphology algorithm can simulate the large deformation of organs in the similarity measurement model, and the principle block diagram of the algorithm is shown in Figure 6a. The Ra and Rb are two disc-shaped structural elements with different radii. The radius of the two elements is denoted by $K(R_{a})$ and $K(R_{b})$.The structure element $\Delta R=R_{a}-R_{b}$ represents the size of the ring region. Set $R_{a}$ as a peripheral structure element, and set $R_{b}$ as the inner structure element. $R_{c}$ represents a structuring element of intermediate size between $R_{a}$ and $R_{b}$. K(ΔR) is the radius of the ring and represents the distance from the center of the ring to the outer contour; therefore, $K(\Delta R)=K(R_{a})$. Y(ΔR) represents the distance from the inner edge of ΔR to the outer contour; therefore, $Y(\Delta R)=K(R_{a})-K(R_{b})$. The relationship between $R_{a}$, $R_{b}$, and $R_{c}$ is illustrated in Figure 6a, where O is the center of the three structuring elements.

Based on the above, we define the following two operations:

(6) $$\begin{array}{l} (f\circ Rab)(x,y)=(f⊕\Delta R)⊙Rc\\ (f\Theta Rab)(x,y)=(f⊙\Delta R)⊕Rc \end{array}$$

The formula for the dual-structured morphological transformation is as follows:

(7) $$\begin{array}{l} WTH^{\prime }(x,y)=f(x,y)-(f⊕\Delta R)⊙Rc\\ BTH^{\prime }(x,y)=(f⊙\Delta R)⊕Rc-f(x,y) \end{array}$$

In the equation, $R_{ab}$ represents our transformation, which is associated with both structuring elements $R_{a}$ and $R_{b}$. As shown in Figure 6(b1–b6), there are different sizes of dual-structure elements for processing, and the images obtained are very different. The purpose of this procedure is to simulate the large deformation of the organ. In addition, sufficient transformation will also increase the anti-interference ability of the model, which is beneficial to enhancing the model’s understanding of the same anatomical structure.

In the process of model training, random affine and the affine transformation are applied to the moving images, including the angle of rotation [−3, 3], displacement [−3, 3], and scaling [8%, +8%]. Then, the moving image is subjected to bistructural morphology (M1ΘQ) and noise processing (M1ΘQ+ε), and ResNet50 is selected as the basic network model. By rotating displacement and other operations, similarity measurement can be ensured to cover spatial position differences, and bistructural morphology and noise processing can be ensured to cover organ distribution differences. Resnet50 deep learning model training can be used to measure pelvic similarity. The second part of Figure 6 shows the basic structure of the network model. The gradient of the residual element of the model can be decoupled into two parts, so the information of the loss function can be propagated backward to any shallower element. At the same time, even if the weight is arbitrarily small, the phenomenon of gradient disappearance can be avoided. The error parameters are predicted by the following formula:

(8) $$\underset{S}{\min}L_{l1}(S)=S_{M1,M2}[{\left\|E(M2,M1\Theta Q+\varepsilon ),(M2-M1)\right\|}_{1}]$$

where M1ΘQ represents bistructural morphological transformation, ε represents random noise, and the original image *M* is transformed by random space twice to obtain *M*1 and *M*2.

The ResNet50 deep learning model is employed to train and obtain similarity measurements for pelvic regions. By rotating displacement and other operations, similarity measurement can be ensured to cover spatial position differences, and bistructural morphology and noise processing can be ensured to cover organ distribution differences. This method is very advantageous for the registration of pelvic parts with large deformation. By combining the high-precision similarity measurement method of bistructural morphology, we can obtain the general multi-modality similarity measurement of pelvic parts with large deformation, which is the key step of accurate registration.

## 3. Results

### 3.1. Datasets

We use clinical CT-MR image data of hospitals to verify the effect of the algorithm. The data include 200 cervical cancer patients, generally IIIB or IIIC, aged 40–72 (average age: 57), to conduct MR Scans on a Philips Achieva 3T scanner (Philips Netherlands) and CT scans on the Siemens Definition AS+ system. The resolution of the MR image is $0.8\times 0.8\times 7\;mm^{3}$, and the resolution of the CT image is $0.97\times 0.97\times 3\;mm^{3}$. All participants were fully informed of the study procedures and agreed to participate in the study.

In the image preprocessing, we first remove the invalid background, such as the bed in the CT image of the patient. This operation is also to extract the regions of interest, that is, only the pelvic region images are retained for each patient. Then, the CT and MR images were unified into the same space size. $1\times 1\times 5\;mm^{3}$ was used in the experiment, and the CT and MR images were unified into the 384×256×32 size to facilitate image input into the model. In all experiments, MR was treated as a moving image and CT as a fixed image. We used data from 160 of these as training and the remaining 40 as testing.

### 3.2. Implementation Details

The MTEF model is implemented using PyTorch with 15,000 iterations, using the Adam optimizer with an initial learning rate set to $1\times 10^{-4}$, multiplied by 0.6 for every 1000 iterations after 5000 iterations. The wavelet basis function of 3D-DWT is db5; during the training of the similarity measurement, the bistructural morphology operators were set with radii of 3, 5, 6, 7, and 9. The training network is implemented on a server with a graphics card type NVIDIA RTX 4090 and an Intel i9-13900KF CPU. In the case of 160 pairs of test data, the training time was 6.7 h, and the registration test time for each CT/MR Image pair was 0.59 s.

### 3.3. Evaluation Index

We illustrate the registration performance of the model using three evaluation indexes, including the Dice Similarity Coefficient (DSC) [31], Average Symmetric Surface Distance (ASSD), and Jacobian determinant (JD) [16]. The following will give a detailed description of the three evaluation indicators.

Dice Similarity Coefficient (DSC): We first calculate the Dice score using label $A^{k}$ of the fixed image and label $B^{k}$ of the moving image to illustrate the algorithm registration effect. Its calculation formula is as follows:

(9) $$DSC=2\cdot \frac{\left|A^{k}\cap {\left(B\circ \phi \right)}^{k}\right|}{\left|A^{k}\right|+\left|{\left(B\circ \phi \right)}^{k}\right|}$$

where ϕ represents the deformation field predicted by the model. If the deformation field is accurate, the overlap rate between *A* and B∘ϕ is high, and the DSC value is close to 1, which also indicates that the registration performance of the model is superior, and vice versa.

Average Symmetric Surface Distance (ASSD): We also evaluated the surface distance between the labels of fixed images and labels of moving images. Its formula is expressed as

(10) $$ASSD=\frac{1}{\left|A_{S}\right|+\left|B_{S}\right|}\left(\sum_{x\in B_{S}}d(x,A_{S})+\sum_{y\in A_{S}}d(y,B_{S})\right)$$

where x and y represent points on the surfaces of $A_{S}$ and $B_{S}$. The smaller the ASSD value, the better the registration effect of the algorithm.

Jacobian determinant (JD): The paper also uses the JD index to describe the topological structure of the deformation field and uses the voxel percentage of $\left|JD\right|\leq 0$ to evaluate the quality of the deformation field. The smaller the value of $\left|JD\right|\leq 0$, the better the predicted deformation field.

### 3.4. Real Cervical Cancer Clinical Data Experiment

**Figure 7:**
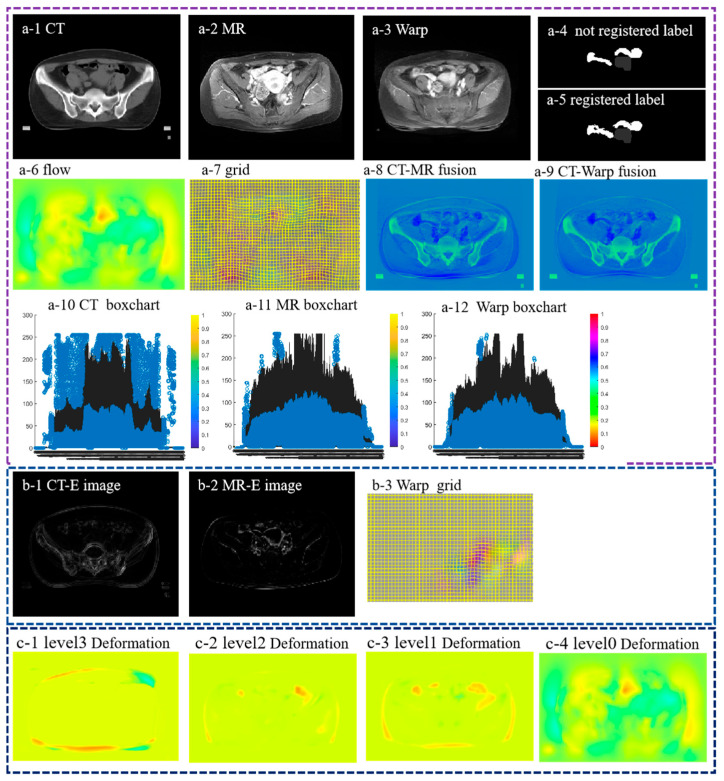
Complete process of algorithm registration: (**a-1**–**a-7**) represents the effect diagram of multi-mode registration, (**a-8**) represents the fusion image of the original CT and MR, and (**a-9**) represents the fusion image of the Warp image and CT, which shows that Warp and CT basically overlap, which will help doctors make use of different information for diagnosis. (**a-10**–**a-12**) represents a boxchart of (**a-1**–**a-3**), respectively. It can be seen that the warp image is distorted towards the fixed image. (**b-1**,**b-2**) represents the enhanced processing of the high-frequency components of CT and MR, respectively, (**b-3**) represents the local deformation field of the model during the local registration process, and (**c-1**–**c-4**) represents the deformation field during the process of level3 to level0.

**Figure 8:**
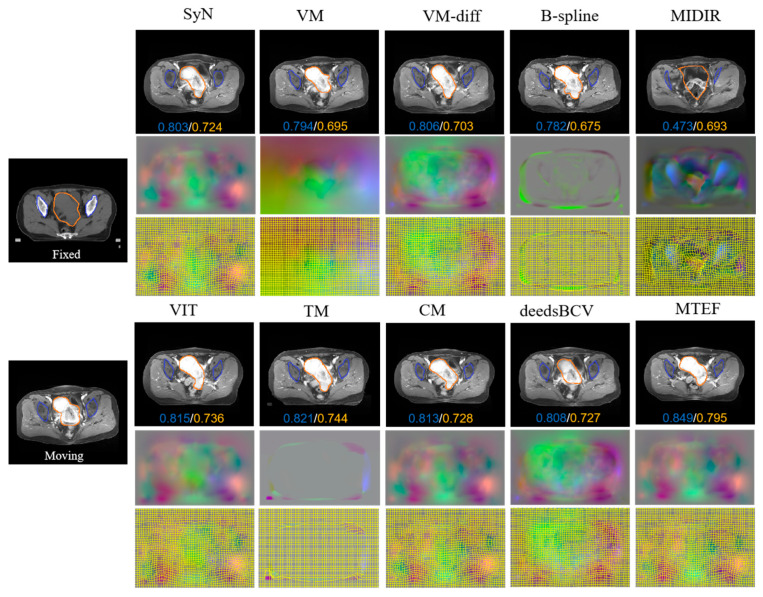
Comparison of registration effects of various algorithms in actual data and deformation field.

**Figure 9:**
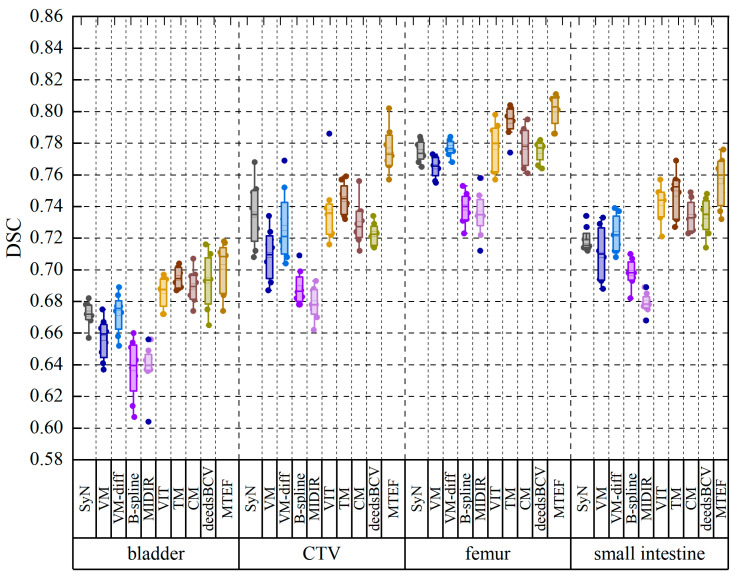
Comparison boxplot of the registration effect of different organs. The second row on the horizontal axis represents different organs of the patient, including the bladder, CTV, femur, and small intestine. The first row represents different algorithms. The vertical axis represents the DSC values obtained by different algorithms.

In this part, we use real clinical data of cervical cancer patients for experiments. Figure 7 shows the registration results of multimodality images and different stages of the model registration process. As shown in Figure 7(a-3), the model obtained warp images that were closer to fixed images, indicating that the model could take into account both global and local registration, and the registration effect achieved the ideal effect (Figure 7(a-5) and Figure 8(a-4) gave the changes of labels provided by the hospital before and after registration). At the same time, as shown in Figure 7(a-6,a-7), it provided the deformation field and deformation mesh generated by the model. The model-obtained deformation field is continuous and smooth. Figure 7(a-8,a-9) show the overlay image of the MR image and CT image and the overlay image of the warp image and CT image. It can also be seen in Figure 7(a-8,a-9) that after registration by our algorithm, the overlap was greatly increased, which indicates that the obtained warp image basically coincides with the fixed image. Figure 7(b-1,b-2) show the input low-frequency information component image and the obtained local registration deformation field during the process of level2 and level3 registration. Figure 7(c-1–c-4) shows the deformation field obtained in the four stages of the model. The registration process follows this rule, from coarse to fine registration. It ensures that the model can take into account local features and global features, and the deformation field obtained is smooth. Based on the intermediate process images obtained by the above model throughout the process, our model achieves satisfactory registration results in large deformation registration work.

### 3.5. Comparisons with the State-of-the-Art Methods

Our test set consists of a total of 20 datasets, with 6 sets designated as L1–L6, and experiments were conducted to compare the MTEF algorithm with state-of-the-art registration algorithms previously presented. It includes four traditional registration methods: SyN [12], Demons [32], B-spline [33], and deedsBCV [34]. It was compared to six deep learning algorithms: VoxelMorph (VM) [19], VoxelMorph-diff (VM-diff) [35], CycleMorph (CM) [14], ViT-V-Net (VIT) [36], TransMorph (TM) [24], and MIDIR [37].

Table 1 shows the results of the comparison with the current advanced registration algorithms. The patients are set to L1–L6. We give the data numbers L1–L6 and the unregistration DSC values for multimodal images in the first column of Table 1. Taking the LI patient as an example, after MTEF model registration, the DSC value of LI is 17% higher than that of the unregistered. We can clearly see that among several algorithms, the mean DSC index of the MTEF algorithm is 5.64% higher than that of the TransMorph algorithm. According to the standard deviation of statistical data, the standard deviation of the DSC index of MTEF is 0.003, which is significantly lower than that of other comparison algorithms, indicating that the algorithm has stable registration performance. The Transmorph algorithm uses a single Swin Transformer as the encoder to extract the image features of different modes and directly generate the deformation field, so the registration in the local complex region does not achieve the ideal effect. In our algorithm, we use a double Swin Transformer to extract different image features, respectively, and adopt a progressive deformation field prediction strategy to achieve a better registration effect. We also compare traditional registration methods; the traditional MIDIR algorithm realizes the registration of multimodal images by generating mode-independent feature descriptors. However, in the task of large deformation, due to the inability to accurately extract local organ features, the deformation field generated by this algorithm is distorted, which leads to the distortion of the organs in the warp image. Moreover, the operation time of the compared algorithms is relatively long. Compared with traditional registration methods, the MTEF algorithm also achieves the highest DSC index in L1–L6 images, which reflects the effectiveness of multi-stage registration strategies that rationally use different components. The CM algorithm also adopts the idea of multi-level registration, but the MTEF algorithm adopts enhanced local information as the input in the high-precision similarity measurement proposed at Level2 and Level3, making MTEF more advantageous than the CM algorithm in large deformation registration work, which can also be seen in several evaluation indicators. In short, the MTEF algorithm can better balance the registration accuracy and the smoothness of the deformation field and capture global and local features at four levels, respectively.

In order to better illustrate the registration performance of the algorithm, as shown in Figure 8, we give a comparison image of the registration effects of various algorithms, and the bottom numbers of the comparison image represent the DSC index for the marked area. The MTEF algorithm obtains registration results that are the closest to the fixed images, especially in the labeled structural regions (orange and blue regions). The registration effect of the MTEF algorithm shows that its enhanced high-frequency component will further improve the ability of the model to extract local features. In addition, from the registration effect of the MIDIR algorithm, it can be found that due to the poor smoothness of the deformation field, the edge of the warp image is discontinuous, and even the internal organs are distorted. Overall, our algorithm achieves the best registration effect and the highest dice index value. In order to better explain the registration performance of the algorithm on each organ, we used the label provided by professional gynecologists to calculate the evaluation parameters of multiple parts. Figure 9 shows the boxplot of the registration effect of different organs between the algorithm and the mainstream registration algorithm. A horizontal line inside the box is the median of the data and indicates the central trend of the data. The length of the boxplot indicates the degree of dispersion of the registered DSC index, as well as the distribution of outliers that can be seen on each individual boxplot. Through the center line and length of the boxplot in Figure 9, we can find the stability and registration performance of the MTEF algorithm. The Dice index of our algorithm exceeds 0.8 in both CTV and femur parts. By combining the statistical indicators in Table 1 with the comparison effect in Figure 8 and the boxplot in Figure 9, we can find that compared with other registration algorithms, this model has the best registration effect and can solve the registration task of large deformation.

## 4. Discussion

### 4.1. Effectiveness of the Proposed SPR-Net Registration Network

In this section, we explore the impact of the SPR-Net registration network on model registration. The SPR-Net module employs two independent Swin Transformer-based encoders and a shared CNN-based decoder. Therefore, by replacing the Swin Transformer-based feature extraction module in the encoder with a CNN-based feature extraction module layer by layer, we investigate the impact of this module on network performance. The standard model is MTEF, and the model with the encoder replaced by CNN is labeled as MTEF-CNN. Additionally, we removed the skip connections from the MTEF model to study their impact, setting this variant as MTEF-NS. From the registration comparison results shown in Figure S2 and the evaluation metrics of various algorithms in Table 2, select one patient’s CT and MR images from the test data and designate them as K1. It is evident that the MTEF model achieves a noticeable registration advantage. This is because, after replacing the encoder with CNN, the model resembles VoxelMorph, which, as we previously discussed, has limitations in handling large deformation complex registration tasks. Even with two-stage registration, it may still struggle to achieve both global and local registration. This also demonstrates that the two independent Swin Transformer encoders in our SPR-Net registration network are better at extracting features from different modalities.

**Figure 10:**
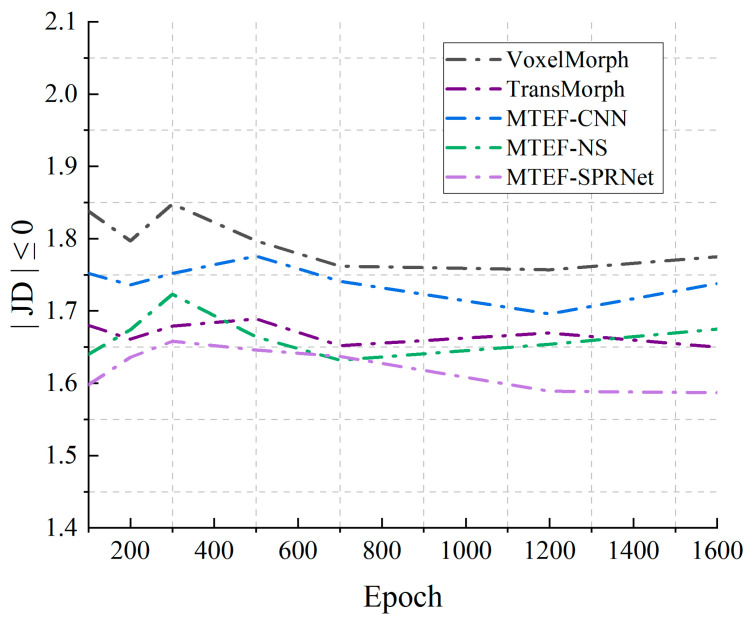
$\left|JD\right|\leq 0$ curve of two advanced registration algorithms and MTEF variants.

Additionally, we explored the impact of skip connections on the registration results. From the registration outcomes, the MTEF model performs better compared to MTEF-NS because the skip connection structure provides more detailed information. It can also be found through the $\left|JD\right|\leq 0$ curve in Figure 10. The deformation field of the MTEF model has the property of differential homeomorphism. In summary, the Swin Transformer encoder structure and skip connections are two critical components of the MTEF model.

### 4.2. Analysis of Similarity Measure Evaluator

**Figure 11:**
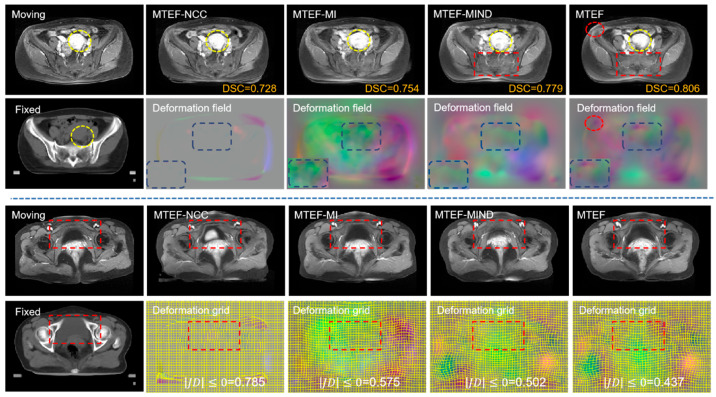
Registration results of MTEF using different similarity algorithms and the resulting deformation field and deformation mesh.

We will explore the influence of similarity measurement in the MTEF model; specifically, we design several variants to explore the registration effect of different similarity operators. These include (NCC), (MI), and (MIND), i.e., the variants are named MTEF-NCC, MTEF-MI, MTEF-MIND, and normal version MTEF (the normal version of the MTEF model uses our proposed deep learning similarity measurement combined with bistructural morphology). Figure 11 shows the registration effect obtained using different variations for two patients. Focusing on the rectangular box can be clearly seen, the results obtained by different similarity operators are quite different, and the effect obtained by MTEF-NCC in the registration work of large deformation is not ideal. MTEF-MIND can handle global deformation well, but local deformation processing is not ideal. In addition, we give the DSC index and JD evaluation index of the deformation field. When other components of the model are the same and only the similarity measurement is different, the MTEF model achieves a DSC value of 0.806, which is higher than that of other similarity measurement variants and 2.7% higher than that of the MTEF-MIND variant, indicating that our proposed bistructural morphology combined with deep learning similarity measurement adopted by the MTEF model can help the model accurately evaluate the error between the warp image and fixed image, thus improving the model registration accuracy.

## 5. Conclusions

Due to the patient’s movement and bladder filling degree, the organs in CT and MR images have nonlinear large deformation, which increases the difficulty of pelvic registration. In this paper, a new unsupervised elastic registration model for large deformation is proposed. First, a deep learning similarity measurement method combined with bistructural morphology is used to train an evaluator for pelvic region registration, which will obtain parameters covering large deformation for loss function. Then, the model adopts wavelet transform to extract different components of the image as model inputs, and we propose a shared pyramid registration network to achieve accurate deformation field prediction. This module employs two independent Swin Transformers as encoders, leveraging the wide receptive field of Transformers to handle large deformation in multimodal registration. In the decoder part, a pyramid feature registration module is used to predict the deformation field, utilizing a coarse-to-fine registration strategy to address large deformation scenarios. The model can effectively use different resolution information to perform four times from coarse to fine registration, thus ensuring the continuity of the deformation field. The experimental results show that our MTEF algorithm has achieved excellent registration performance in the actual clinical cervical cancer image registration work, and the algorithm can solve the problem of the complex and large deformation of multi-modality images of pelvic cavity.

## Figures and Tables

**Table 1:** Registration evaluation indexes of various advanced algorithms.

Datasets /Methods	Metrics	Demons	SyN	VM	VM-Diff	CM	B-Spline	deedsBCV	MIDIR	VIT	TM	MTEF
L1 (0.683)	DSC	0.698	0.724	0.703	0.712	0.735	0.687	0.693	0.709	0.743	0.749	0.803
	$\begin{array}{l} \left|JD\right|\\ \leq 0 \end{array}$	0.726	0.438	1.934	1.946	1.623	0.669	0.723	1.241	0.687	0.592	0.395
	ASSD	1.106	0.917	1.013	1.054	0.845	1.158	1.086	0.931	0.854	0.859	0.812
L2 (0.687)	DSC	0.679	0.706	0.713	0.709	0.728	0.694	0.681	0.713	0.735	0.756	0.806
	$\begin{array}{l} \left|JD\right|\\ \leq 0 \end{array}$	0.732	0.574	1.844	1.896	1.472	0.673	0.746	1.157	0.632	0.613	0.413
	ASSD	1.096	0.958	1.062	1.057	0.873	1.135	1.065	0.935	0.851	0.851	0.826
L3 (0.692)	DSC	0.694	0.719	0.708	0.714	0.716	0.702	0.695	0.707	0.741	0.767	0.802
	$\begin{array}{l} \left|JD\right|\\ \leq 0 \end{array}$	0.739	0.552	1.852	1.842	1.541	0.676	0.738	1.236	0.667	0.597	0.386
	ASSD	1.114	1.097	1.088	1.062	0.881	1.132	1.061	0.932	0.847	0.843	0.817
L4 (0.695)	DSC	0.683	0.713	0.715	0.702	0.725	0.683	0.701	0.702	0.729	0.769	0.807
	$\begin{array}{l} \left|JD\right|\\ \leq 0 \end{array}$	0.724	0.521	1.796	1.873	1.546	0.664	0.731	1.241	0.654	0.622	0.381
	ASSD	1.058	1.065	1.071	1.047	0.873	1.128	1.059	0.946	0.843	0.846	0.824
L5 (0.697)	DSC	0.687	0.705	0.694	0.719	0.734	0.697	0.695	0.714	0.736	0.763	0.811
	$\begin{array}{l} \left|JD\right|\\ \leq 0 \end{array}$	0.726	0.637	1.863	1.925	1.568	0.685	0.728	1.226	0.658	0.573	0.406
	ASSD	1.067	9.751	1.084	1.066	0.874	1.136	1.066	0.937	0.856	0.835	0.813
L6 (0.689)	DSC	0.691	0.721	0.723	0.685	0.727	0.696	0.708	0.711	0.738	0.771	0.804
	$\begin{array}{l} \left|JD\right|\\ \leq 0 \end{array}$	0.741	0.624	1.847	1.866	1.473	0.679	0.735	1.237	0.649	0.588	0.392
	ASSD	1.063	1.062	1.076	1.082	0.868	1.141	1.069	0.933	0.852	0.839	0.822

**Table 2:** Registration evaluation indexes of different algorithms and MTEF variants.

Datasets	Metrics	SyN	VM	VM-Diff	MIDIR	CM	B-Spline	TM	MTEF-CNN	MTEF-NS	MTEF-SPRNet
K1	DSC	0.753	0.718	0.735	0.746	0.727	0.742	0.761	0.732	0.794	0.805
	ASSD	1.322	1.749	1.435	1.535	1.697	1.254	1.128	1.557	0.946	0.732

## Data Availability

The research data are stored in an institutional repository and will be shared upon request to the corresponding author.

## Supplementary Materials

The following supporting information can be downloaded at: https://www.mdpi.com/article/10.3390/bioengineering11121304/s1, Figure S1. (a): Swin Transformer extracts feature maps at different stages of the image and computes self-attention within local D windows, then passes the generated features to subsequent CNN decoders. (b): Self-attention computation based on the shifted window design; Figure S2. a-1 to a-5 shows the registration effect of the three variants, the second row is the corresponding deformation field, and the third row is the overlapping image of Warp image and CT obtained by the corresponding algorithm.
