# Supplementary material for: Complex Large-Deformation Multimodality Image Registration Network for Image-Guided Radiotherapy of Cervical Cancer
Source: Bioengineering (Basel). 2024 Dec 23;11(12):1304. doi: 10.3390/bioengineering11121304 (PMC11726759; doi:10.3390/bioengineering11121304)
Supplement: Supplementary file 1 [file bioengineering-11-01304-s001.zip › bioengineering-3334774-supplementary.pdf]

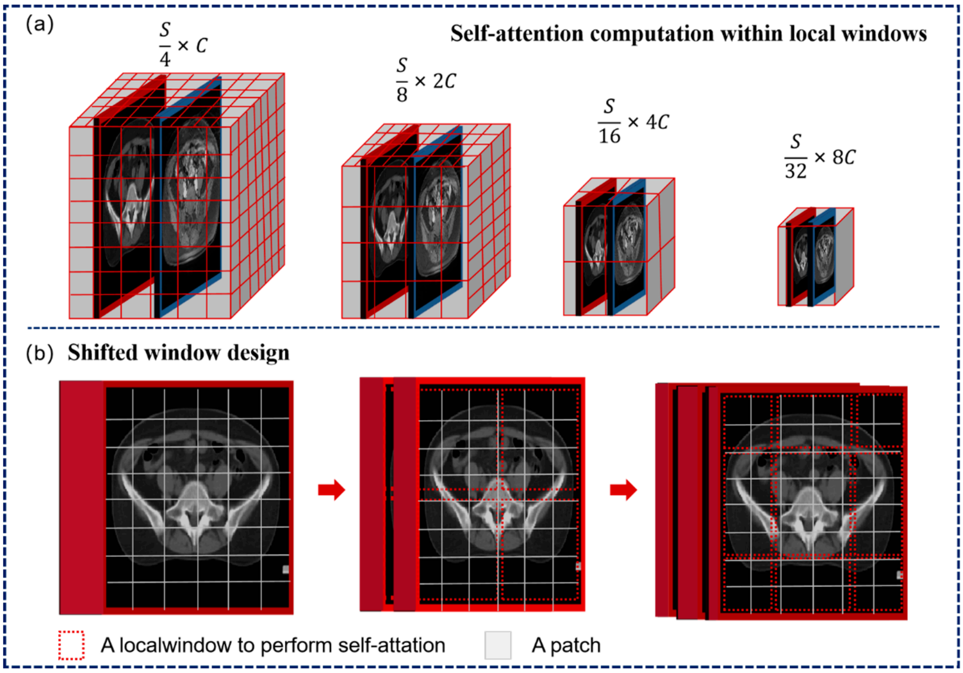

Figure S1. (a): Swin Transformer extracts feature maps at different stages of the image and computes self-attention within local D windows, then passes the generated features to subsequent CNN decoders. (b): Self-attention computation based on the shifted window design.

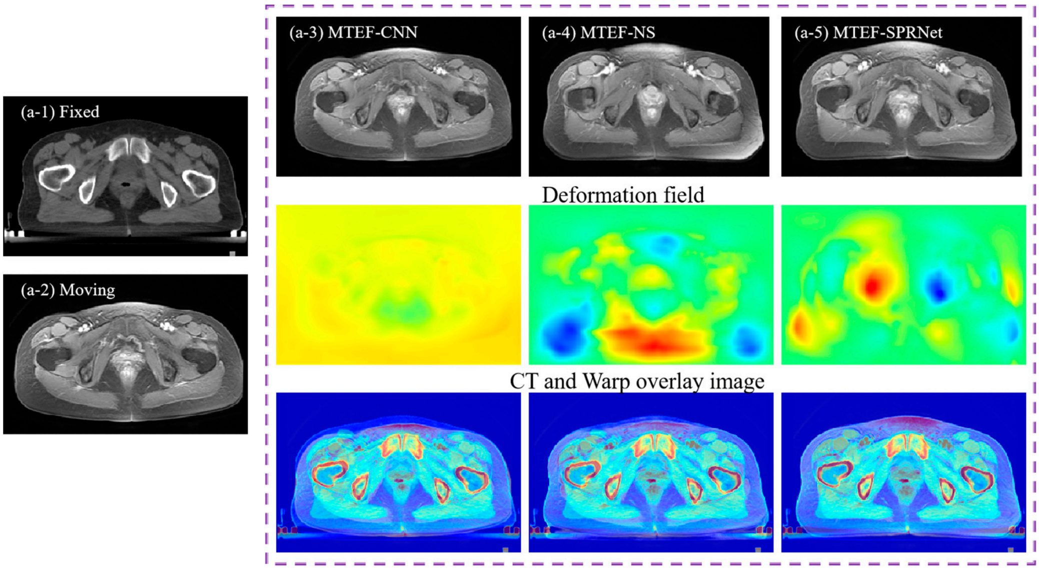

Figure S2. a-1 to a-5 shows the registration effect of the three variants, the second row is the corresponding deformation field, and the third row is the overlapping image of warp image and CT obtained by the corresponding algorithm.
